# Mechanical Forces in Fetal Lung Development: Opportunities for Translational Research

**DOI:** 10.3389/fped.2013.00051

**Published:** 2013-12-25

**Authors:** Juan Sanchez-Esteban

**Affiliations:** ^1^Department of Pediatrics, Alpert Medical School of Brown University, Providence, RI, USA

**Keywords:** mechanical forces, lung development, tracheal ligation, growth factors, heparin-binding EGF-like growth factor

Pulmonary hypoplasia secondary to congenital diaphragmatic hernia, oligohydramnios, etc., is an important cause of neonatal morbidity and mortality. In fact, pulmonary hypoplasia is the most common finding (up to 26%) in neonatal autopsies (1). Moreover, more than 20,000 babies are born every year in the United States before 27 weeks of gestation (canalicular stage of lung development). These disorders have in common an incomplete development of the lungs. In addition to the risk of death, these conditions can also cause severe respiratory distress at birth and serious long-term morbidities (2). Currently, the management is primarily supportive and there are not specific treatments designed to accelerate the development of the lungs.

The lungs are unique in that their growth and development depends primarily on extrinsic factors and specifically on mechanical forces (3–7). During gestation, the epithelium of the lung secretes fluid creating a constant distension pressure in the lumen of the lung of approximately 2.5 mm Hg (8). Moreover, the fetus makes episodic breathing movements (FBM) starting in the first trimester and increasing in frequency up to 30% of the time by birth (9). Whereas there is agreement on how much pressure is generated inside the fetal lung by fluid secretion, the same does not apply to the change in length experienced by the lung with each FBM. Part of this controversy is due to the complex and variable changes in thoracic dimensions and intraluminal pressures generated by FBM (10). During non-accentuated periods of FBM, the intraluminal pressures may only decrease by 2–3 mm Hg. However, this pressure can decrease up to 10–15 mm Hg during accentuated periods of FBM (11, 12). It has been suggested that distension of the fetal lung generated by FBM is negligible, as they generate very little tidal movement of fluids (10, 13). Based on changes in thoracic shape observed during FBM and assuming the cone shape of the thorax and spherical shape of the distal potential airspaces, it has been speculated that FBM might result in repetitive changes in distal lung surface area of about 5% (14). Experiments in fetal lung explants and fetal type II epithelial cells have shown that a stimulus of that magnitude results in cell proliferation (15) and differentiation (5). In either case, it is clear from experimental animals that drainage of lung fluid volume (16) or abolition of FBM (17, 18) lead to lung hypoplasia. Therefore, both tonic hydrostatic distension and cyclic mechanical deformation provide physical signals necessary for normal fetal lung development. However, the mechanisms by which the fetal lung senses these mechanical signals to promote development are not well characterized.

Tracheal ligation, as a mechanism to increase the pressure inside the lung to accelerate development, has been used in animal models (19) and in humans fetuses affected by congenital diaphragmatic hernia (20). However, this method has a high rate of complications such as preterm labor, premature rupture of membranes and even death (21), and limitations for its use in other forms of pulmonary hypoplasia. Therefore, a different approach to this problem is to investigate how mechanical forces promote lung development and use that information to stimulate lung development.

**Figure 1:**
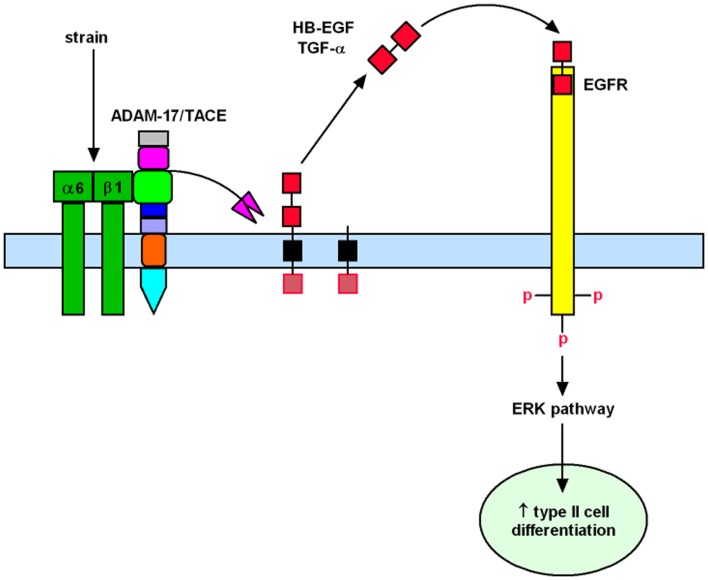
**Mechanistic model to show how mechanical forces promote differentiation of fetal type II epithelial cells via release of soluble growth factors HB-EGF and TGF-α with subsequent binding and activation of the EGFR and ERK signaling pathway**.

Past investigations in fetal lambs have shown that lung fluid composition after tracheal ligation was critical to promote lung development, since acceleration of growth and differentiation was not observed when lung fluids were replaced with normal saline (22, 23). These studies suggest that increased intratracheal pressure after tracheal ligation releases soluble factors that are important for lung development. This hypothesis is supported by previous *in vitro* studies from our laboratory in which fetal type II epithelial cells isolated during the canalicular stage of lung development were exposed to mechanical strain mimicking mechanical forces in lung development. Our data showed that mechanical strain cleavages and releases the soluble mature forms of epidermal growth factor receptor (EGFR) ligands HB-EGF and TGF-α (24, 25). Release of these soluble factors bind and activate the EGFR via autocrine or paracrine signaling and promote differentiation of type II cells via the ERK signaling pathway (26) (Figure 1).

The identification of soluble factors released by mechanical forces that are important for normal lung development could lead to novel avenues to accelerate lung development. Potential translational research applications would be prenatal administration to fetuses affected by pulmonary hypoplasia secondary to congenital diaphragmatic hernia or oligohydramnios or fetuses with borderline viability (22–24 weeks) and at risk for delivery. Another theoretical application would be postnatal administration via the endotracheal tube. This is just an example of how the information obtained from these *in vitro* mechanistic studies could have the potential for clinical applicability. However, before considering their use in humans, rigorous experiments in animal models are required to demonstrate the effectiveness of these therapies and the lack of untoward side effects.
